# Response to issues on GM agriculture in Africa: Are transgenic crops safe?

**DOI:** 10.1186/1756-0500-4-388

**Published:** 2011-10-08

**Authors:** Ademola A Adenle

**Affiliations:** 1 United Nations University Institute of Advanced Studies (UNU-IAS), 6F International Organisations Centre, Pacifico Yokohama, 1-1-1 Minato Mirai Nishi-ku, 220-8502, Yokohama, Japan; 2 National Graduate Institute for Policy Studies (GRIPS), 7-22-1 Roppongi, Minato-ku, Tokyo 106-8677, Japan

## Abstract

The controversies surrounding transgenic crops, often called Genetically Modified Organisms (GMOs), call for a need to raise the level of public awareness of Genetic Modification (GM) technology in Africa. This should be accomplished by educating the public about the potential benefits and risks that may be associated with this new technology. In the last 15 years, GM crop producing countries have benefited from adoption of this new technology in the form of improved crop productivity, food security, and quality of life. The increased income to resource-poor farmers is a key benefit at the individual level especially as most countries using this technology are in the developing world, including three African countries (South Africa, Burkina Faso and Egypt). Despite clear benefits to countries and farmers who grow GMOs, many people are concerned about suspected potential risks associated with GMOs. This sparks debate as to whether GM technology should be adopted or not. Given the concerns regarding the safety of GMO products, thorough scientific investigation of safe application of GMOs is required. The objective of this paper is to respond to the issues of GM agriculture in Africa and some of the issues surrounding the adoption of GM crops between developed and developing countries. In this article, I analyse relevant papers relating to the adoption of GM technology particularly in developing countries including the few African countries that have adopted GM crops. The issues discussed span a wide range including: safety; potential benefits and risks; disputes between the United States of America (USA) and the European Union (EU) over adoption of GM crops with a focus on Africa continent. This article is concluded by summarising the issues raised and how GM technology can be adopted for agricultural development in Africa.

## Discussion

### Why must GMOs be given a chance in Africa?

A growing body of evidence-based reports continue to document increased crop yields, increased farm income, and health and environment benefits associated with GM crops [1-10]. In 1996, when GM crops were first officially commercialised, six countries planted a total of 1.7 million hectares of these crops [9]. By 2010 this had grown to 148 million hectares in 29 countries, of which 19 are from the developing world. This 87-fold growth makes GM the fastest crop technology to be adopted in the history of modern agriculture. Of 15.4 million farmers that planted GM crops in 2010, over 90% (14.4 million) are resource-poor farmers in developing countries, including three African countries (Burkina Faso, South Africa and Egypt), that benefited from the adoption of GM crops [9]. For example, almost 100,000 farmers in Burkina Faso cultivated GM cotton on 260,000 hectares in 2010 with 126% increase from 115,000 hectares planted in 2009. In terms of contribution to the economy of Burkina Faso, it was estimated that a benefit of over US$100 million per year could be generated based on almost 30% increase in yield [11]. Additionally, close to a 50% reduction in insecticides sprays may be realised, thereby saving fossil fuels and lowering greenhouse gases emission, hence fighting against climate change. South Africa, the first and biggest producer of GM crops in Africa, has benefitted from growing GM crops over the past decade. They are the only African country among the five principal countries (also India, Argentina, Brazil and China) producing GM crops, with 63 million hectares planted in 2010 alone. The economy of South Africa has benefited a great deal from the adoption of GM technology and it has been reported to have enhanced farm income from GM maize, GM soybean and GM cotton by US$156 million in the period 1998 to 2006, with 2006 alone estimated to be US$67 million [12]. Egypt was the first North African country to grow GM crops commercially in 2008 with 700 hectares of GM maize planted and the Egyptian government planning to increase the hectarage of GM crops in future [13].

In contrast to the documented benefits of GM crops, there are few documented cases of potential health effects [14] or economic drawbacks [15,16] to GM crops. However, the issues of potential risks of GMOs continue to be raised, particularly in Europe, and the media is being used to attract attention to the question of whether GMO products are safe. For example, the "Flavr Savr" tomato is a genetically modified tomato that has altered DNA to delay ripening, thereby prolonging shelf-life [17]. It was the first GM food to be authorised for human consumption in the United States and received broad coverage by the media even though it is not a poison or a food known to cause illness. A GM soybean that contained a Brazil nut allergen [18] is arguably the only incident that got more attention from the media when GM crops were first commercialized. The intense media coverage resulted in cessation of further development of this particular variety due to potential health risks. In contrast, another incident that attracted media attention was that of a scientist that claimed a genetically engineered potato caused a depressed immune response in rats [19], but subsequent investigation showed that the experiment was scientifically flawed.

It should be noted that eating conventional foods is not risk free as they are known to contain allergies. For example, there was no known allergy when kiwifruit were introduced into the European and United States markets in the 1960s, but the fruits are now known to cause allergic reactions [20]. GM technology is like any other new technology and has its merits and drawbacks. Over the period of time that commercially available GM foods have been produced, no studies have indicated that GM foods are less safe than traditional counterparts. Although merit may be given to concerns of unintended gene flow from genetically engineered agricultural products, further studies are required to establish the reality and/or scope of this and other potential environmental risks due to GMOs. Unintended adverse effects of GMOs on non-target species (e.g. butterflies) have been reported to be similar to what currently exists in traditional agricultural products [21]. While there are other individual claims that GMOs could pose health risks to human beings, most of these findings are not peer-reviewed in international scientific journals or by any officially recognised standard. Rather, these are individual works that are being promoted on websites or by non-governmental organisations (NGOs). Unfortunately, when issues like these are raised and debated, many people condemn GMOs outright based upon unverified sources and also fail to see if there are advantages associated with the application of GMOs.

Several international regulatory bodies including the World Health Organisation (WHO), the Food and Agriculture Organisation of United Nations (FAO) have concluded that there is no scientific evidence that the application of GM technology has resulted in substantial human health effects or environmental problems [22,23]. For example, a British government report released by a panel of experts found out that no verifiable ill effects have been reported from consumption of GM food products and that the risks to human health are very low for current GM crops on the markets [23]. In spite of the lack of evidence for potential adverse effects of GMO production, most European countries do not grow GM crops as precautionary principle to ban GM crops has been adopted in European legislation. This precautionary principle can be invoked when: "potentially dangerous effects deriving from a phenomenon, product or process have been identified, and ...scientific evaluation does not allow the risk to be determined with sufficient certainty" because of the insufficiency of the data or their inconclusive or imprecise nature" [24]. In other words, this precautionary principle can be stated as: action can be taken against potential hazards if the scientific information does not prove lack of risk. While the use of this precautionary principle may be based upon a lack of scientific assessments of the risks of adopting GM crops, it has been criticised due to lack of assessment of economic, social and ethics aspects of GM adoption [25]. Additionally, this precautionary principle has been interpreted differently in international treaties, court decisions, academic literature and government policy reports [26]. This has effectively made it difficult to refer to the precautionary principle as a single or collective term.

While the precautionary principle has been presented in several versions, there is no clear existing scientific consensus within the scientific community with regards to any of the risks that may have been found or associated with GMO products. But the question remains: Are there books or peer-review journals that have recorded the number of illnesses or deaths as a result of consumption of GMO products in any part of the world? It is unfortunate that many people and organisations including government officials in Africa see GM technology as a poison that must be avoided and not to be introduced into their decaying traditional farming systems. Some of these problems can be attributed to lack of awareness and education on the application of modern biotechnology among Africans. For example, it was recently reported that scientists have not effectively communicated the benefits and risks of modern biotechnology to the Kenyans [27], hence leading to a high level of public concern and protests against GM crops. The failure of the scientists to play a role in public education might be attributed to negligence on the part of Kenyan government or may reflect a lack of communication and/or confidence between government and scientists over GMOs issues. These issues should be addressed and the public must be educated on both the potential economic benefits of GM crops and also the general lack of scientific evidence for health related issues when GM crops are consumed.

### Africa must be saved from hunger

Given the high level of poverty, malnutrition, hunger, food security problems, and low agricultural productivity in Africa, advanced technology like GM technology has the potential to offer solutions to some of these problems. However, the controversy over the use of GM technology remains one of the biggest threats in adopting this new technology. The current regulatory approaches of the United States of America (USA) and the European Union (EU) can play an important role over adoption of GM technology in developing countries, particularly in Africa [22,28,29]. For example, the USA can allow commercial release of GMO products based upon standard tests such as allegenicity, digestivity and toxicity without any regulatory barrier, whereas in the EU, GMO products can be stopped based upon scientific uncertainty alone without any associated evidence of risks and sufficient testing according to the so called ''precautionary principle''.

The precautionary principle has become a political tussle, without clearly defined concepts, among giant nations [22]. This tussle has a serious impact on the African continent as to whether to adopt GM technology or not. Reports have shown that developing countries, including Africa, consider their trade relationships with the EU before adopting GM crops due to the fear of losing this market [30,31]. As a result, African governments tend to exhibit a ''go slow approach'' toward the adoption of GM technology. This is often reflected in delayed approval of biosafety laws required to grow GM crops, with the majority of African countries lacking a functional biosafety system [32]. An attempt to ascertain what causes delay in biosafety law approval has attributed the delay to political lobbying, change of administration, and the issue not being a high priority for the policymakers [33].

Why should we fold our arms when many people are starving, and children are dying as a result of malnutrition, in Africa and there is an innovative technology to solve part of the problem? For example, when about 14 million people in Southern African countries were faced with a food crisis due to drought, the food aid provided by the USA was rejected by Zimbabwe and Zambia simply because the maize was GM. People in these nations were left to suffer from starvation as a result. Although Zimbabwe reversed its decision after diplomatic intervention from pro-GM countries, Zambia placed an outright ban on GM maize from USA, stating potential health risks associated with the food. Severe drought in East Africa, Kenya, has resulted in recent import of GM maize due to the shortage of maize. However, many people, led by anti-GM activists, protested against the importation of GM maize as they feared it would contaminate the soil and could be harmful to the health of the citizens. Fortunately, the importation of GM maize was cleared by the Kenyan Cabinet after a proper safety assessment and clearance from the National Biosafety Authority.

Apart from constraints such as inadequate infrastructure and facilities, most channels of influence that are associated with adoption of GM technology in Africa come mainly from EU, and their tough regulatory precautionary principle can be a major threat to commercial export sales when African countries start producing GM crops [22,28,31]. This is not only restricted to African countries but other developing countries are also suffering a similar fate. Recently, the Director of the Beijing Genomics Institute strongly advised that EU should change its current GMO policies that hinder China and other developing countries from enjoying full benefits of these new agricultural technologies [34]. European Greenpeace is arguably the most organised group in the world that is campaigning against GMOs; they played a key role in influencing Zambian government officials to reject any GMO products coming into the country [22]. Any attempt to allow GMO products could mean that organic agricultural product export sales to the EU may be lost. While the USA considers GMO products safe enough for human consumption, after going through compulsory testing with their conventional counterparts, the EU regulatory approach is probably not in best interest of GMO products [29]. While no clear evidence has been established against GM crops, the EU remains sceptical about the use of GM crops despite spending more than $425 million on studying the safety of GM crops over the past 25 years [35].

Moreover, it is unclear what roles international organisations such as the World Trade Organisation (WTO) and the Convention on Biological Diversity (CBD) are playing in mediating the disputes between the USA and the EU as there does not seem to be cooperation on contending issues. Rather, it appears that multiple factors (political, economic, institutional, legal, historical and cultural) have combined together in a manner that undermines potential cooperation and mutual benefit between the two parties [36]. This lack of cooperation affects many poor countries in decision making on these new agricultural technologies. Why is there such a divide or lack of common interest to promote and encourage the fastest technology in the history of modern agriculture to fight poverty, malnutrition, hunger and food insecurity in Africa? Answering this question is beyond the scope of this article. Perhaps, this question can be answered as more knowledge about GM technology becomes available and when assessing the global benefit of this new technology becomes a reality. Current evidence shows that GM technology has a great potential to improve agricultural productivity and farmers' livelihood in developing countries. Therefore, GM technology must be allowed to play a part in alleviating hunger and poverty in Africa.

### Safety is paramount: America and others leading the way

It is important to ensure the safety of all citizens that consume GM foods either in Africa or any part of the world. Not many countries are using GMOs in agricultural production around the world due to health and environmental concerns associated with the application of GMOs. The USA is one of the few developed countries growing GM crops on a commercial basis. More than 70 percent of some foods in the USA contain GMOs [37], yet no American citizen or others that have consumed their foods have been confirmed seriously ill or dead as a result of GMO consumption.

In view of the fact that no global consensus has been reached over completely safe application of GMOs and as the need to regulate or control GMO products become more important, the USA can be a good example for Africa and the rest of the world. The USA appears to be a lead country in safe application of GMO products with the United States Department of Agriculture (USDA) reporting that 93 percent of all soybeans, 78 percent of all cotton, and 70 percent of all corn grown in the USA in 2010 were genetically modified to be herbicide tolerant [38]. In fact, USA regulatory bodies have determined that GMO foods are "substantially equivalent" to conventional foods. Before any GMO products are released for human consumption, they are compared with traditional counterparts in a variety of chemical, genetic, biochemical, compositional, nutritional and environmental tests as well as comparison with known allergens [39]. The USA Food and Drug Administration (FDA) monitors every product made up of GMOs for comprehensive safety and environmental testing. Other GM producing countries, including Canada, Australia, China, and Argentina are carrying out similar tests to ensure that GMO products are safe for human consumption.

Although, the USA's FDA cannot be compared with majority of food & drug regulation departments in African countries which are without sophisticated equipment to do thorough testing and analysis, some of these countries have effective regulatory system with excellent track records in food safety. With support from developed countries, it should be possible to increase local capacity for testing in Africa and this should ensure safe delivery of GMO products while taking necessary precautions. The USA and other countries are good examples to follow when it comes to adoption of GM technology. After all, it is how this technology is used that determines whether it is risky, good, or beneficial. Therefore, as with any technology, continued evaluation of GM technology in terms of safety and benefits will be important as more knowledge becomes available. African governments can learn to follow the same procedures that other countries are already employing to ensure the safety of their citizens when GMO products become available in Africa.

### Working towards common interest: embracing the Gene Revolution in Africa

Over four decades the Green Revolution was implemented and African countries were bypassed without any documented benefits. In contrast, Asian countries have benefited in terms of improved seeds, chemicals, pesticides and inorganic fertilizers. This has brought about phenomenal successes in boosting crop production in corn, wheat and rice [40]. The emergence of the Gene Revolution is assumed to play a significant role whereas the Green Revolution was not able to deliver in Africa. The slow embracement of the Gene Revolution in Africa, particularly GM technology, is the lack of access for beneficiaries such as farmers and scientists to GM crops. This lack of access is, at least in part, due to international influence against GMOs and this international influence remains fundamental to the successful adoption of GM technology in Africa.

There are a lot of issues affecting the adoption of GM technology. These require a concerted effort from African governments and developed countries to ensure successful transfer of the technology to the beneficiaries. In this regard, one of the major challenges which can delay access to GM crops is dealing with intellectual property right (IPR) issues. GM technology is currently being applied in production of the main commercial crops such as cotton, corn and soybean. Clearly this creates the largest profit for the biotech companies (e.g. Monsanto). However, it is difficult to access the technology in developing countries as biotech companies are often reluctant to invest in the development of this new technology in Africa. Most of the developing countries (especially Africa) do not have strong and effective laws to protect IPR and the legal systems can be very poor, this may partially explain why biotech companies are not attracted to African countries. Given the problem of the IPR logjam, one can imagine a lengthy period before innovative technologies that have potential benefits for orphan crops (e.g. millet, cassava, potato, sweet potato, yam, banana, cowpea and sorghum) can be realized as GM crops in African countries. Some hope may be on the horizon as the African Agricultural Technology Foundation (AATF) is working towards facilitating and promoting partnerships between the public and private sector to ensure that resource-poor farmers have free access to agricultural technologies including GM technology. Efforts are being made by AATF in partnership with other institutions towards developing GM crops that can benefit African farmers. For example, African scientists in collaboration with other scientists in developed countries are carrying out research on orphan crops such as GM banana, GM cassava and GM cowpea with field trials being carried out in Uganda and Nigeria respectively.

Other than IPR issues, the adoption of GMOs is strongly influenced by developed countries particularly the USA and EU. The USA and EU clearly demonstrate unequal levels of trust of the safe application of GMOs. This can be an obstacle for adopting this new technology in Africa. While the European influence in Africa seems stronger than the American influence, particularly in trade partnership, economic and political power should not stand in the way of new technology. Apart from foreign aid that comes from the EU, the majority of African countries have closer cultural ties with Europeans (stemming from the colonial era) than Americans. Thus, the majority of African countries are more likely to stick to the views and practices of European systems [22]. However, such practices or systems must not dominate agricultural policies in Africa. Because agricultural practices are a matter of life and death in Africa, they must be sustained and improved to feed the poor continent. The developed countries like the USA, the EU and others should work in common interest of delivering GM technology that has potentials to improve agricultural productivity, health and food security in Africa.

### Africa must take action

Agriculture plays a significant role in the economy of African countries in terms of gross domestic product (GDP), promoting international trade, industrial development and creation of job opportunities. Therefore, there is an urgent need to improve agricultural production. GM technology can be a part of the solutions in cases where traditional methods of farming have been less efficient. The potential benefits of GM crops toward alleviating poverty and hunger, improving agricultural productivity, health, food security and creating a friendly environment cannot be overemphasised. The adoption of GM technology is at its initial stages in Africa and is currently faced with several constraints such as lack of infrastructures, inadequate human resource capacity, poor education, biosafety regulation, intellectual property rights and many others. A concerted effort from developed countries including international organisations must be put in place to ensure that Africa benefits from this new technology. African governments must also be involved in solving these problems themselves and they should come up with coherent strategy to adopt modern biotechnology including educating the public, farmers and government institutions, the media and private companies, and to increase understanding of GM technology. Part of the strategy must include adoption of common policies and a regional platform through which African governments can engage in dialogue and develop a common biotechnology regulatory approach. Africa might pay a huge price in many years to come if the continent continues to depend on outsiders before making decisions that determine their future. Europeans are well fed and may not necessarily require GM technology to boost their crop productions, but African farmers need fast technology that can solve part of their agricultural problems.

## Competing interests

The author declares that they have no competing interests.

## Authors' contributions

AAA solely designed and reviewed all the papers used for developing the manuscript.
